# Caudate lobe resections: a single-center experience and evaluation of factors predictive of outcomes

**DOI:** 10.1186/1477-7819-11-220

**Published:** 2013-09-05

**Authors:** Prejesh Philips, Russell W Farmer, Charles R Scoggins, Kelly M McMasters, Robert CG Martin

**Affiliations:** 1 Department of Surgery, Division of Surgical Oncology, University of Louisville, 315 E Broadway, Suite# 311, Louisville KY 40202, USA

**Keywords:** Caudate, Hepatocellular carcinoma, Metastatic colorectal, Cholangiocarcinoma

## Abstract

**Background:**

Despite the increasing frequency of liver resection for multiple types of disease, caudate lobe resection remains a rare surgical event. The goal of this study is to review our experience and evaluate possible predictors of adverse outcomes in patients undergoing caudate lobectomy.

**Methods:**

We reviewed a 1,900-patient prospective hepato-pancreatico-biliary database from January 2000 to December 2011, identifying 36 hepatectomy patients undergoing caudate lobe resection. Clinicopathologic characteristic and outcome data were compared using chi-square, *T*-test, ANOVA, Kaplan-Meier, and Cox regression analysis. Primary endpoints were the incidence and severity of complications, and secondary endpoints were blood loss, hospital stay, and transfusion requirements. Patients were also divided in two groups with group A being patients operated on before December 2007 and group B after 2007. We compared the demographics, risk factors, complication rates, and operative details between the two groups.

**Results:**

Thirty-six patients underwent caudate lobe resection for cholangiocarcinoma (47.2%), metastatic colorectal cancer (36.1%), hepatocellular carcinoma (8.3%), or benign disease (8.3%). Nine patients (29%) had additional liver resection. Median overall survival (OS) was 21 months. Complications occurred in 52.7% (19/36) of patients with a median grade of 2. Tobacco abuse was associated with an increased risk of operative complications (73.3% vs. 38.9%, *p* = 0.03). Prior history of cardiac disease was associated with a higher complication rate (87% vs. 42%, *p* = 0.03). Neoadjuvant chemotherapy, biliary procedures, hepatitis, and prior major abdominal surgery were not predictive of complications. Major complication was also predicted by the volume of RBC transfusion (2.7 vs. 4.1 units, *p* = 0.003). In our subgroup analysis of the patients undergoing surgery before and after 2007, the two groups were well matched based on age, comorbidities, and risk factors. The complication rates and rates of high-grade complications were similar, but blood loss (600 ml vs. 400 ml, *p* = 0.03), inflow occlusion time (Pringle time 12.6 vs. 6, *p* = 0.00), and hospital stay (9.5 vs. 7 days, *p* = 0.01) were significantly lower in group B.

**Conclusions:**

With appropriate patient selection, caudate lobe resection is an effective component of surgery for hepatic disease. Tobacco use and prior cardiac history increase the risk of complications.

## Background

In the last 2 decades there have been dramatic improvements in the morbidity, mortality, and long-term outcomes in hepatic surgery. Improved surgical techniques, new instrumentation, and low central venous pressure (CVP) surgery have led to improved outcomes as well as increased the number of patients eligible for curative hepatectomy for a number of diseases [1]. This is especially true for malignancy with subsequent improved survival for patients undergoing hepatectomy for metastatic colorectal cancer (MCRC) [2], hepatocellular carcinoma (HCC) [3], and cholangiocarcinoma [4]. This in turn has led to an increase the number of successful hepatectomies for longer-term disease control.

Despite the increase in hepatectomy rate, the rate of caudate lobe resection remains low because of a combination of factors. The unique anatomic location and specific anatomic features of the caudate lobe render resection technically challenging [5-7]. In addition, the blood supply of the caudate further complicates resection because of the difficulty of vascular isolation with ongoing dialogue as to the best surgical technique and true vascular anatomy of this region [8,9]. Special care must be taken regarding the caudate lobe’s close proximity to the middle hepatic vein, retrohepatic caval attachments, and left-hepatic arterial supply. As a result of these factors, modern caudate lobectomy has only recently been initially described [10]. Since that time, caudate lobectomy has increased in frequency and has been shown to be an effective treatment for HCC [11], MCRC [12,13], and cholangiocarcinoma [4]. However, the vast majority of data are reported from smaller series or non-Western populations [14].

Given the current paucity of data, this study attempts to review the available data following caudate resection from our tertiary referral center. The goal was to review our data and delineate pathologic and clinical factors potentially associated with adverse outcomes for patients undergoing caudate lobectomy.

## Methods

We performed a review of a 1,900-patient prospective hepato-pancreato-biliary database spanning from January 2000 to December 2011. Patients were included if they underwent caudate lobectomy for any indication. Patients were included if they had concurrent hepatectomy or other organ resection. Patient selection and operative technique were the same as has been presented previously [15-19]. Patients were excluded for incompleteness of data or age <18 years. The university IRB approved this study. We evaluated potential outcome predictors with one primary endpoint: development of postoperative complications. Secondary endpoints assessed were requirement of blood transfusion, hospital stay, and the number of blood products transfused. We attempted to analyze recurrence, but the data for this analysis were not available/incomplete in the majority of patients. Complications were graded according to CTCAE v4.0 criteria [20]. A severe-grade complication was defined as one that was of grade ≥3, with a maximum complication grade of 5 (i.e., requiring intervention, either operative or radiologic).

In order to analyze the impact on the outcome over different periods in time, patients were divided into two groups according to the year of operation, namely before 2007 and after. We chose 2007 as an arbitrary cutoff point based on our change in practice of liver transection technique transitioning from a crush-clamp and clip model to a simultaneous precoagulation and transection device. With our increasing experience with laparoscopic liver resections, this was also around the time that we abandoned routine use of inflow control (Pringle) and an increasing number of patients had received modern chemotherapy (including FOLFOX). Eighteen patients each underwent caudate lobe resections before and after December 2007. We similarly compared outcomes including complications, high-grade complications, operative times, and intraoperative blood loss between the two groups.

Data were analyzed using chi-square, Fisher’s exact, independent *T*, Kaplan-Meier, and Cox regression tests utilizing SPSS (Chicago, IL, USA). A *p*-value < 0.05 was considered statistically significant.

## Results

Thirty-six patients underwent caudate lobectomy over this 11-year period reviewed. Of these, the majority was for MCRC (*n* = 16, 44.4%) or cholangiocarcinoma (*n* = 14, 38.9%), with the remainder for either HCC or benign disease (*n* = 3, 8.3% each). Tweenty-two were male (61.1%). Median age at presentation was 61, with 30.5% having concomitant cardiopulmonary disease. Tobacco abuse was present in 15 (41.7%) patients, and among tobacco smokers, the median usage was 21 pack years (IQR 12–31). History of alcohol abuse was seen in seven patients (19.4%).

Complications were seen in 19 out of 36 patients (52.7%), of whom 8 (22.2%) were high grade, requiring additional intervention. Factors associated with complications as well as with high-grade complications were analyzed (Table 1) using univariate and multivariate analysis. The most common complication was ileus (10.8%). Significant hepatic complications were infrequent: two patients developed biliary complications, two bilomas, and one bile leak. In addition, only two patients suffered from perioperative liver insult, neither of whom had permanent liver failure.

**Table 1:** Factors associated with complications

**Factor**	**Any complication, *n = *19**	**High-grade complication, *n = *8**	***P-*value^**
**Alcohol abuse**	5 (13.9%)	1 (2.8%)	0.25/0.5
**Cardiac h/o (*n *= 8 22.2%)**	**7** (**19**.**4**%)	**2** (**5**.**6**%)	**0**.**03**/**0**.**5**
**Tobacco abuse (*n *= 15 41.7%)**	**11** (**30**.**6**%)	4 (11.1%)	**0.04**/0.4
**Hepatitis (*n *= 3 8.3%)**	2 (5.6%)	0	0.9
**Left lobectomy**	4 (11.1%)	0	0.1/0.1
**Right lobectomy**	6 (16.7%)	2 (5.6%)	0.09/0.1
**Colectomy**	6 (16.7%)	5 (13.9%)	0.17/0.2
**Transfusion**	9 (25%)	2 (5.6%)	0.3/0.1
**Number of units pRBC (median)**	2.7	**4**.**1**	0.1/**0.003**
**Number of lesions removed (mean)**	2.2	3.5	0.04/0.8

^*p* value: Significance using univariate analysis: Fisher’s exact and chi-square test for categorical and independent group *t* test for numerical variables. Numbers in bold are for significant values. The first value is the *p* value for all complications, and second value is for high-grade complications.

Tobacco abuse was associated with an increased risk of postoperative complications (73.3% vs. 38.9%, *p* = 0.04). A history of prior cardiac disease (*n* = 8) was associated with an increased overall complication rate (87% vs. 42%, *p* = 0.03) but not high-grade complications, hospital stay, or mortality. Multivariate analysis also showed an association of increased complication rates with tobacco use (OR = 1.7, *p* = 0.05 with CI 0.2-2.7) and a history of cardiac dysfunction (OR = 1.9, *p* = 0.03 with CI 0.7-4.1). Among the other comorbidities, three patients (8.3%) had a history of viral hepatitis, but only one patient had ascites/cirrhosis at the time of resection. Other medical comorbidities did not predict outcomes for morbidity or mortality.

With regards to presenting complaints, the majority (*n* = 13, 34.7%) were related to biliary obstruction or stasis, with 14 (38.9%) requiring preoperative biliary decompression. Thirteen (36.1%) patients received neoadjuvant chemotherapy with 19 (52.7%) receiving adjuvant therapy. Neoadjuvant therapy was also not associated with an increased risk of complications (9 had complications, *p* = 0.3).

Operative times ranged from 80–360 min (median 180 min, IQR 140–237.5). Median intraoperative blood loss was 450 ml (IQR 300–800); central venous pressure (CVP) at the time of parenchymal transection was 2 (IQR 1–5). In ten patients (27.8%) Pringle inflow vascular control was used. Blood transfusion was given in 20 patients (55.6%). Vascular reconstruction was done in five patients (2 HCCs, 2 MCRCs, 1 cholangiocarcinoma) with three (9.7%) portal vein reconstructions and two caval reconstructions. Resection margins were microscopically positive (R1) in the final specimen in 22.2% (*n* = 8). The majority of these positive margins occurred in patients with cholangiocarcinoma with 75% of positive margins occurring in this group (*p* = 0.041).

Isolated caudate resection was performed in 22 patients (61.1%). Concurrent, non-caudate hepatectomy was performed in 14 patients; 7 each had a left or right lobectomies (19.4% each), including 7 patients who underwent a trisegmentectomy (19.4%). Patients with a diagnosis of mCRC were compared with patients with cholangiocarcinoma and were noted to have a significantly shorter hospital stay (8.3 vs. 13.3 days, *p* = 0.010), significantly lower blood transfusion requirements (37% vs. 62%, *p* = 0.05), and significantly lower positive margin rates (6.8% vs. 42.8%, *p* = 0.03). They were also found to have lower overall complication rates (50% vs. 71%, *p* = 0.07), shorter operative times (173 min vs. 203 min, *p* = 0.4), and less blood loss (540 ml vs. 626 ml, *p* = 0.21), but this did not reach statistical significance.

One perioperative death was seen in an 87-year-old woman undergoing caudatectomy for palliation, 4 years status post-sigmoidectomy, for CRC receiving 10 units of perioperative RBCs. Subsequently, she developed complications from a percutaneous gastrostomy and was never able to recover from this, succumbing to multiorgan system failure within a few days.

The median number of lesions (*p* = 0.2) removed did not predict the development of a complication. Complication was also unaffected by the performance of common bile duct resection (*p* = 0.1); the interaction of bile duct resection as well as the diagnosis of cholangiocarcinoma was not a confounder in this analysis (*p* = 0.7). The need for transfusion did not predict complication (42% vs. 58%, *p* = 0.3), but trended toward a higher rate of high-grade complications with the mean number of units transfused far greater in those patients developing a high-grade complication (2.8 vs. 6.6, *p* = 0.007).

Predominant pathologic findings of the normal peritumoral hepatic parenchyma had the majority of inflammatory findings: inflammatory 36%, fibrosis 11%, steatosis 17%, and normal 25%. Inflammatory pathology trended toward increased operative times (165 vs. 202 min, *p* = 0.1) and blood loss (430 vs. 690 ml, *p* = 0.06), but this difference did not reach statistical significance.

With regards to the subset of the 22 patients who only underwent isolated caudate lobectomy, 9 each (41%) were for MCRC and cholangiocarcinoma. Their age and distribution of comorbidities were similar to those of the larger cohort. Complications were seen in 9 of 22 patients (41%), of which 6 (27.3%) were high grade, requiring additional intervention. Tobacco use (62.5% vs. 28.6, *p* = 0.08) and a history of prior cardiac disease (67% vs. 35%, *p* = 0.07) were associated with an increased overall complication rate, which was not statistically significant. Operative time (median 160, IQR 120–232), intraoperative blood loss (median 350, IQR 225–500), CVP (median 3, IQR 2–6), patients receiving blood transfusions (*n* = 14, 63.6%) and the number of transfusions in patients receiving them (2, IQR 0–3), R1 resections (*n* = 5, 22.7%), and overall hospital stay (6.5 days, IQR 4.7-10) were similar to those of the larger cohort.

We then analyzed the potential differences between two subgroups of patients undergoing operations on (group A) or before (group B) December 2007 (Table 2). There were 18 patients in each group, and these patients were similar in median age (64.1 and 60.3 years), cardiopulmonary comorbidities, diagnosis, type of procedure performed, and number of tumors removed (1.67 vs. 1). Median OS was 19 months for group A and 21 for group B (*p* = 0.8).

**Table 2:** Subgroup temporal analysis: peri- and postoperative analysis

**Factor**	**Group A: before 2007 (18)**	**Group B: after 2007 (18)**	***P *(univariate analysis)**
**Complications**	10 (55.5%)	9 (50%)	0.9
**High-grade complications**	2 (11.1%)	2 (11.1%)	1
**Mean number of tumors**	1.67	1.93	0.5
**Operative time (median)**	180	192	0.38
**Blood loss**	600 ml	400 ml	**0**.**03**
**OS**	19	21	0.8
**Blood transfusion**	7	13	0.09
**Blood units**	1.0	1.9	**0**.**01**
**CVP parenchyma**	3	2	0.08
**Pringle time**	12.6 min	6.3 min	**0**.**00**
**Hospital stay**	9.5 days	7 days	**0**.**01**
**Positive tumor margins**	3 (16.6%)	5 (27.8%)	0.7

Complication rates and high-grade complication rates were similar in both groups with group A having a 5% higher complication rate (55% vs. 50% for B, *p* = 0.9). The two groups were analyzed for factors associated with an increased complication rate (Table 2). Median operative times were similar (group A: 180 min and group B: 192 min, *p* = 0.38). Median intraoperative blood loss in group A was 600.3 ml versus 400 ml in group B, which was statistically significant with a *p* value of 0.03. However, the transfusion requirements and total number of units received were not significantly different. Of note, the median CVP at parenchymal transection was 3 in group A vs. 2 in group B (*p* = 0.08). Median length of stay in the hospital was significantly longer at 9.5 days before 2007 as compared to 7 days after 2007 (*p* = 0.01). A similar number of margins were positive before and after 2007 (3 vs. 5, *p* = 0.6), and a similar number of patients had undergone a prior colectomy.

## Discussion

Caudate lobe resection is a challenging operation with evolving techniques. The caudate lobe can be involved as the primary liver lesion of an isolated metastatic site. In view of its intimate relationship with the inferior vena cava and the portal vein, presence of multiple short caudate veins, and posterior location, it is difficult to resect. Caudate lobe resection can be done in isolation or combined with left or right lobectomies depending on the disease burden. In view of the paucity of data in the Western literature, we reviewed our experience over the last decade. The majority of caudatectomies performed were either for MCRC or cholangiocarcinoma. Tobacco use and a prior history of cardiac disease were independent predictors of morbidity on uni- and multivariate analysis. Neoadjuvant chemotherapy, prior colectomy, and blood transfusions had no significant effect on complication rates. Development of high-grade complications was higher in patients undergoing right lobectomies with caudatectomies, but this did not reach statistical significance. The performance of prior colectomy did not impact the complication rate or the incidence of high-grade complications. Similarly, the need for blood transfusions did not correlate with higher complication rates in our study. We also looked at the pathologic findings of the peri-tumoral hepatic parenchyma and found that inflammatory pathology trended toward increased operative times and blood loss. This could be a reflection of the native background hepatic disease or a reflection of prior adjuvant therapy such as hepatotoxic chemotherapy or arterial embolization therapy.

Our subgroup analysis comparing patients undergoing caudate resections before 2007 to those after was carried out to gain a better insight into the temporal trends of performance and factors affecting them over time. This arbitrary division at December 2007 was made because it was approximately midway in the study period and divided the study population into two equal and relatively well-matched groups. The two groups were similar in comorbidities, age, and primary diagnosis. We noted an improvement in complication rates, hospital stays, and operative times; however, these did not reach statistical significance. Blood loss was significantly lower in the latter group, and this could be attributed to a lower parenchymal transection CVP in the later years (which showed a lower trend in the second half but was not statistically significant). We attribute this improvement in these parameters to a combination of factors including more uniform use of improved energy devices for parenchymal transection and improved implementation of controlled CVP. The total Pringle time in patients requiring it was significantly lower in the latter group, and this along with the overall more expedient outcome (lower operative times, shorter stays) is once again probably due to a confluence of many factors. Our experience over the years modulated by the advent of laparoscopic liver resections has led to a decreased use of inflow occlusion (Pringle maneuver). This was in part due to better parenchymal transection technology with less blood loss and adaptation to intraparenchymal inflow and outflow control with stapling as an evolution to minimally invasive liver resections.

There has been a paucity of studies in the Western literature on the outcomes and predictors associated with caudate lobectomy. This article is an attempt to fill the lacuna and better understand the factors affecting the selection, distribution, and morbidity of caudate resections. The majority of reports have been similar in scope, between approximately 20 to 50 patients undergoing caudate resection [11-13,21-23]. The median operative time within these series varies from 230–400 min, significantly higher than our median of 180 min (Table 3). Whether this is due to technique or variability of the disease pattern cannot be readily discerned because of the variance among the data, but it is likely that both contribute. Similar comparison can be made between the amount of intraoperative blood loss, with several series reporting an average EBL of 1,200 ml [23], 1,590 ml [24], and 1,683 ml [22], respectively, whereas the EBL in our series was lower (450 ml). Interestingly, few studies reported their rate of perioperative complications, and fewer still graded the complication rates. The rate of major complications we experienced, again, was similar to that seen by others [12]. Hawkins et al. [25] also showed that major vascular resection was predictive of poorer overall survival (OS); this was not evaluable in our series with so few (*n* = 5) undergoing portal or caval reconstruction.

**Table 3:** Caudatectomy series comparison

**Author**	***n***	**Pathology**	**OR time**	**EBL**	**%Morbidity**
Philips	36	Mixed	180	450	52.7%
Hawkins [25]	150	Mixed	317	800	55.3%
Liu [11]	114	HCC	NA		18%
Morise [26]	8	CRC	315	1,325	12.5%
Sakoda [22]	12	HCC	400	1,683	8.3%
Sarmiento [27]	19	Mixed	211	760	5%
Wen [21]	11	HCC	NA	300	54.5%
Zuo [28]	16	HCC	255	740	NA

Mixed = combined MCRC, HCC, and cholangiocarcinoma. EBL = estimated blood loss in ml.

The novelty of these series, including our own, is tempered by their retrospective nature as well as potential selection bias. Specifically, our own series included only one patient with significant cirrhosis/ascites so as be classified as a Child’s class B cirrhotic, making this study not generalizable to the entire population of patients with severe hepatic disease or malignancy. Also the number of patients with a history of hepatitis is underrepresented. However, this bias will be inherent regardless given the nature of caudate resection, as one would be remiss in subjecting a patient with advanced hepatic disease to this type of resection knowing the potential for postoperative morbidity and mortality. The pathology in our study was mixed such that meaningful data for long-term survival analysis and recurrence rates would be hard to interpret. Also, we acknowledge that different biologies requiring caudate resections led to differences in operative techniques and outcomes. Cholangiocarcinoma often requires concurrent bile duct resection as well as more preoperative biliary decompression, which changes the morbidity profile of these resections. Similarly, colorectal metastasis and hepatocellular carcinoma in the caudate lobe tend to have greater vascular invasion, as was seen in our study. Despite these limitations, this study provides meaningful insight into the safety of caudate resections as well as delineation of the risk factors. Modern hepatic surgery is ever evolving with improvement in perioperative mortality and morbidity over the years. This trend is reflected in our series. Better transections techniques, improved preoperative planning adjuncts, and ever evolving and improving surgical techniques inherent to complex surgery such as caudatectomies are reflected in our temporal subgroup analysis. None of the patients in this series underwent laparoscopic caudate lobe resections, which recent publications suggest is being performed in high-volume centers and is feasible [29,30].

## Conclusion

In summary, caudate lobectomy is a safe and viable means of operative treatment for benign or malignant hepatic disease, though it remains technically demanding. Complications in caudatectomy are associated with more advanced disease and tobacco use. Mortality is similarly associated with more advanced disease as well as the presence of inflammatory findings on hepatic biopsy. Further analysis is needed to determine which factors are truly associated with disease-specific outcomes for caudate resection given the paucity of descriptive literature.

## Competing interests

The authors declare that they have no competing interests.

## Authors’ contribution

RCM, RF, KMM and CSR provided the study concept. Study design was by PP, RF, RCM. Data acquisition was by PP, RCM, RF, KMM and CSR. Quality control of data and algorithms were devised by PP and RCM. Data analysis and interpretation was performed by PP, RF, RCM. Statistical analysis was by PP and RCM. Manuscript preparation and editing was by PP, RCM. Manuscript was reviewed by PP, RF, KMM, CSR and RCM. All authors read and approved the final manuscript.
